# Oxygen-Enhanced MRI Detects Incidence, Onset, and Heterogeneity of Radiation-Induced Hypoxia Modification in HPV-Associated Oropharyngeal Cancer

**DOI:** 10.1158/1078-0432.CCR-24-1170

**Published:** 2024-08-09

**Authors:** Michael J. Dubec, James Price, Michael Berks, John Gaffney, Ross A. Little, Nuria Porta, Nivetha Sridharan, Anubhav Datta, Damien J. McHugh, Christina J. Hague, Susan Cheung, Prakash Manoharan, Marcel van Herk, Ananya Choudhury, Julian C. Matthews, Geoff J.M. Parker, David L. Buckley, Kevin J. Harrington, Andrew McPartlin, James P.B. O’Connor

**Affiliations:** 1 Division of Cancer Sciences, University of Manchester, Manchester, United Kingdom.; 2 Christie Medical Physics and Engineering, The Christie NHS Foundation Trust, Manchester, United Kingdom.; 3 Clinical Oncology, The Christie NHS Foundation Trust, Manchester, United Kingdom.; 4 Clinical Trials and Statistics Unit, The Institute of Cancer Research, London, United Kingdom.; 5 Radiology Department, The Christie NHS Foundation Trust, Manchester, United Kingdom.; 6 Division of Psychology, Communication and Human Neuroscience, University of Manchester, Manchester, United Kingdom.; 7 Bioxydyn Ltd, Manchester, United Kingdom.; 8 Centre for Medical Image Computing, Department of Medical Physics and Biomedical Engineering, University College London, London, United Kingdom.; 9 Biomedical Imaging, University of Leeds, Leeds, United Kingdom.; 10 Division of Radiotherapy and Imaging, The Institute of Cancer Research, London, United Kingdom.; 11 Radiation Oncology, Princess Margaret Cancer Center, Toronto, Canada.

## Abstract

**Purpose:**

Hypoxia mediates treatment resistance in solid tumors. We evaluated if oxygen-enhanced MRI–derived hypoxic volume (HV_MRI_) is repeatable and can detect radiotherapy-induced hypoxia modification in human papillomavirus–associated oropharyngeal head and neck squamous cell cancer.

**Experimental Design:**

A total of 27 patients were recruited prospectively between March 2021 and January 2024. HV_MRI_ was measured in primary and nodal tumors prior to standard-of-care (chemo)radiotherapy and then at weeks 2 and 4 (W2 and W4) into therapy. Two pretreatment scans assessed biomarker within-subject coefficient of variation and repeatability coefficient (RC). Cohort treatment response was measured using mixed-effects modeling. Responding lesions were identified by comparing HV_MRI_ change with RC limits of agreement.

**Results:**

Oxygen-enhanced MRI identified hypoxia in all lesions. The HV_MRI_ within-subject coefficient of variation was 24.6%, and RC limits of agreement were −45.7% to 84.1%. A cohort median pretreatment HV_MRI_ of 11.3 cm^3^ reduced to 6.9 cm^3^ at W2 and 5.9 cm^3^ at W4 (both *P* < 0.001). HV_MRI_ was reduced in 54.5% of individual lesions by W2 and in 88.2% by W4. All lesions with W2 hypoxia reduction showed persistent modification at W4. HV_MRI_ reduced in some lesions that showed no overall volume change. Hypoxia modification was discordant between primary and nodal tumors in 50.0% of patients.

**Conclusions:**

Radiation-induced hypoxia modification can occur as early as W2, but onset varies between patients and was not necessarily associated with overall size change. Half of all patients had discordant changes in primary and nodal tumors. These findings have implications for patient selection and timing of dose de-escalation strategies in human papillomavirus–associated oropharyngeal carcinoma.

*See related commentary by Mason, p. 5503*

**Translational Relevance**

Tumor hypoxia reduces the effectiveness of radiotherapy, chemotherapy, and immunotherapy, leading to poorer outcomes. Recent studies have suggested that patients with human papillomavirus–associated oropharyngeal carcinoma may benefit from radiation dose de-escalation guided by the persistence of hypoxia. We show here that oxygen-enhanced MRI can map the variable onset and duration of radiotherapy-induced hypoxia modification, demonstrating that early scanning could identify which patients will benefit from dose de-escalation. Half of all patients with both a primary and nodal tumor had discordant hypoxia modification, in which only one lesion changed with therapy, emphasizing that personalized therapeutic approaches should consider all measurable disease rather than one target lesion. Hypoxic volumes were not simple surrogates of overall tumor volume, confirming the value of measuring hypoxia as an outcome variable. Collectively, these data support using oxygen-enhanced MRI in larger studies to evaluate dose de-escalation.

## Introduction

Hypoxia is a feature of nearly all solid tumors, including head and neck squamous cell carcinoma (HNSCC; ref. 1). The presence and extent of hypoxia indicate both poor prognosis and resistance to radiotherapy (2), chemotherapy (3), targeted therapies, and immunotherapy (4). Imaging can be used to identify whether patients have hypoxic tumors prior to therapy and to quantify the extent, spatial, and temporal variation of hypoxia (5). This has the potential to assist adaptive radiotherapy. Specifically, there is evidence that identifying hypoxia modification early in therapy can guide dose de-escalation in patients with human papillomavirus (HPV)–associated oropharyngeal carcinoma (6–8) to reduce normal tissue toxicity, or dose escalation in patients with HNSCC with persistent hypoxic subregions (9).

Questions remain over when and how imaging should be used to assist management of patients with HNSCC with tumor hypoxia. PET data from patients with HNSCC have shown that high pretreatment hypoxic volume indicates an increased risk of treatment failure (10). Furthermore, because imaging can monitor serial evolution of hypoxia during treatment, multiple independent PET studies have reported that persistence of hypoxic subvolumes during chemoradiotherapy at 1 to 5 weeks may be most predictive of treatment failure (11–13) in patients with a variety of HNSCC subtypes, including HPV-associated oropharyngeal carcinoma. Persistence of hypoxia following 1 week of chemoradiotherapy has been shown to occur in up to 50% of patients with HPV-associated oropharyngeal carcinoma in studies using PET hypoxia imaging (7).

These data suggest that hypoxic volume may be a more useful clinical biomarker than hypoxic fraction, which is the favored biomarker used in preclinical studies (14), and may assist in future adaptive radiotherapy strategies. However, few studies have compared the hypoxia modification observed in both primary tumor and nodal metastases following treatment, or the timing of these changes (15). This is potentially important to determine optimum radiotherapy planning.

Oxygen-enhanced MRI (OE-MRI) is a noninvasive hypoxia imaging technique, with spatial resolution comparable to PET, that can be performed on standard MRI systems (16). In the majority of OE-MRI studies, subjects inhale high-concentration oxygen (i.e., 100% O_2_ gas) while *T*_1_-weighted MRI is acquired. Well-oxygenated tissues exhibit increases in the longitudinal relaxation rate (*R*_1_) when breathing the hyperoxic gas, whereas hypoxic regions have no significant change in *R*_1_ (17). Changes in tissue *R*_1_ (termed Δ*R*_1_) and related OE-MRI biomarkers that measure MRI hypoxic fraction (HF_MRI_) and hypoxic volume (HV_MRI_) have identified and mapped hypoxia in animal models (18–21) and may predict outcome (22). The same OE-MRI biomarkers have detected hypoxia modification from chemoradiotherapy in animal models and in patients with lung cancer (23).

Previous work has shown that OE-MRI is feasible in patients with HNSCC (24). The primary purpose of this study was to determine if OE-MRI-derived HV_MRI_ was repeatable in patients with HPV-associated oropharyngeal carcinoma. The second purpose was to characterize the onset, duration, and within-patient variation of radiotherapy-induced changes in HV_MRI_ and other OE-MRI–derived biomarkers including Δ*R*_1_ and HF_MRI_ as well as lesion whole tumor volume (WTV).

## Materials and Methods

Patients with p16-positive oropharyngeal carcinoma were recruited into a prospective clinical trial (ClinicalTrails.gov NCT03646747) with institutional review board approval (REC 18/NW/0563), in accordance with the Declaration of Helsinki. Patients provided written informed consent and were scanned at The Christie NHS Foundation Trust between March 2021 and January 2024.

Inclusion criteria were patients aged 18 years or older with biopsy-proven oropharyngeal carcinoma who were due to start definitive (chemo)radiotherapy with no metastatic disease outside of the local neck nodes. Biopsy samples showing strong, diffuse cytoplasmic and nuclear staining in >70% of tumor cells were classified as p16-positive, where p16 immunohistochemistry (IHC) represents a surrogate marker of previous HPV infection (25). Patients were also required to have Eastern Cooperative Oncology Group performance status 0 to 2, creatinine clearance (Cockcroft–Gault) ≥30 mL/minutes, be able to lie comfortably for up to 60 minutes, no history of severe COPD, and be willing and able to consent to the study. Exclusion criteria were previous cancer therapy, pregnancy, history of gadolinium allergy, or contraindication to MRI scanning.

MRI was performed on either a 1.5 T diagnostic MR (Philips Ingenia MR-RT, Philips Medical Systems, Best) or a 1.5 T MR Linac system (Elekta Unity, Elekta, Stockholm), as both systems perform OE-MRI equivalently (24). Patients underwent MR imaging prior to radiotherapy (baseline 1, BL1), followed by scans at week 2 (W2) and/or week 4 (W4) into commencing radiation treatment. A subset of patients had two pretreatment scans to enable repeatability assessment, baseline 0 and 1 (BL0 and BL1) at 1 week apart. All patients received radiotherapy prescriptions between 55 Gy in 20 fractions (#) to 70 Gy in 35#. Concurrent platinum-based therapy was given to eligible patients, following international practice.

MRI sequences were harmonized between the two MR systems (Supplementary Fig. S1; Supplementary Table S1 for receive coil information and additional sequence parameter details). Patients were set up in the treatment position on a flat table top, without thermoplastic shell, on both MR systems. Imaging was acquired in the transverse plane covering the neck region (11.2 cm craniocaudal coverage), and MR imaging sequences included:

1 *T*_2_-weighted fast-spin echo multislice anatomical imaging with Dixon-based fat suppression.
2 *T*_1_ relaxation time measurement [3D inversion recovery turbo field echo (IRTFE), 3 × 3 × 5 mm^3^, inversion prepulse delay times (TI) = 100, 500, 800, 1,100, and 4,300 ms].
3 Dynamic OE-MR acquisition using the same 3D IRTFE sequence with TI = 1,100 ms, 91 measurement timepoints, and temporal resolution = 12 seconds. Gases were delivered at 15 L/minutes through a high-concentration, nonrebreather oxygen mask (EcoLite, Intersurgical Ltd.). Initially, medical air was given (dynamic timepoints 1–25, 5 minutes), followed by 100% oxygen (dynamics 26–70, 9 minutes), finally returning to medical air (dynamics 71–91, 4 minutes).
4 DCE-MRI acquired using a 3D *T*_1_-weighted fast field echo Dixon sequence (3 × 3 × 5 mm^3^) with IV contrast agent injection [Dotarem, 0.2 mL/kg (0.1 mmol/kg) at 3 mL/seconds with 20 mL saline flush], delivered by contrast power injector (Experion, Bayer) at the eighth of 45 dynamic measurement timepoints.
5 Postcontrast 3D *T*_1_-weighted fast field echo acquisition with spectral fat saturation to assist lesion delineation.

Image processing and analysis were carried out using MATLAB (R2018a, MathWorks, RRID: SCR_001622). Motion correction and registration were carried out using Elastix (v5.0.1, https://elastix.lumc.nl; refs. 26, 27). Primary tumors (T) and regional neck metastatic nodal (N) lesions were delineated on postcontrast *T*_1_-weighted images by an HNC clinical oncologist (7 years’ experience) using JIM software (JIM 6, Xinapse Systems, RRID: SCR_009589). Lesion WTV was calculated in cm^3^.

*T*_1_ maps (units ms) obtained on air breathing (21% O_2_) were derived by nonlinear least squares fitting to the IRTFE signal [S(TI)] acquired at the five TI values. This sequence employed TR > 5*T*_1_ and very short TE, such that the TR and TE terms can be ignored and the nonlinear fit estimation of *T*_1_ is

$$S\left(\text{TI}\right)=\;S_{0}\left|1-2\text{λexp}\left(\frac{-\text{TI}}{T_{1}}\right)\right|$$ (A)

in which *S*_0_ is the equilibrium signal, TI is the inversion prepulse delay time, and *λ* is the inversion efficiency parameter. *S*_0_, *T*_1_, and *λ* were set as free parameters during fitting. Measurement of native *T*_1_ permitted estimation of *R*_1_(*t*) (= 1/*T*_1_(*t*)) (units seconds^−1^) during the dynamic OE-MRI acquisition as

$$R_{1}\left(t\right)=\;-\frac{1}{\text{TI}}\ln\left\{\frac{1-\;\left[\frac{S\left(t\right)}{S_{\text{air}}}\left(1-2\lambda \exp\left(\frac{-\text{TI}}{T_{1}}\right)\right)\right]}{2\lambda }\right\}$$ (B)

in which *S*(*t*) is the raw signal intensity and *S*_air_ is the median of *S*(*t*) measurement timepoints 2 to 25, acquired during the air phase. Per-voxel change in *R*_1_ was calculated by Δ*R*_1_ = *R*_1,O2_ − *R*_1,air_ (28), with *R*_1,air_ as the median of *R*_1_(*t*) measurement timepoints 2 to 25, acquired during the air phase and *R*_1,O2_ as the median of *R*_1_(*t*) over timepoints 60 to 70, acquired at the end of the period of 100% oxygen inhalation. Lesion Δ*R*_1_ was calculated as the median of voxel-wise Δ*R*_1_ values per lesion.

Next, dynamic OE-MRI identified voxels in which the signal intensity enhanced significantly (*P* < 0.05) between air (timepoints 2–25) and 100% oxygen (timepoints 60–70) breathing phases using a paired *t* test (29). Similarly, DCE-MRI data identified voxels in which signal enhanced significantly (*P* < 0.05) between pre- (timepoints 2–8) and postcontrast (timepoints 15–45) measurements. Voxels which enhanced on DCE-MRI but not on OE-MRI data were classed as hypoxic (19), enabling calculation of HF_MRI_ (unitless) and HV_MRI_ (units cm^3^; Supplementary Fig. S2). Voxels that enhanced on both OE-MRI and DCE-MRI were classed as normoxic, allowing estimation of the normoxic volume (NV_MRI_; cm^3^). Voxels that did not enhance on DCE-MRI or OE-MRI were classed nonperfused (19). Quality control steps included checking for protocol adherence, acceptable native *T*_1_ value, and absence of motion following motion correction.

No formal sample size calculation was performed in this exploratory study. OE-MRI derived parameters Δ*R*_1_, HF_MRI,_ HV_MRI_, NV_MRI_, and WTV were assessed for distribution normality using a Shapiro–Wilk test. The within-subject coefficient of variation (wCV) and repeatability coefficient (RC), with limits of agreement (LOA), were calculated, and where data were not normal, data underwent log transformation (30). Back-transformed data provided asymmetric upper (RC_U_) and lower (RC_L_) LOA on RC.

Cohort change was assessed using mixed-effects modeling to account for multiple lesions with patient clustering as a random effect; analysis was performed using STATA (BE 17.0, StataCorp, RRID: SCR_012763; ref. 31). Multiple comparisons between baseline (BL) and W2 and/or W4 were considered. In cases in which patients had two BL visits, the mean of the parameter values for the two BL visits (BL0 and BL1) was taken. Per-lesion level analysis was assessed if change in parameter values from BL exceeded the calculated RC LOA, and in doing so, these were considered real change (30). *P* < 0.05 was considered significant in all statistical assessments.

### Data availability

Data were generated by the authors and available on request because an appropriate recognized platform for sharing the study data does not exist.

## Results

### OE-MRI is well-tolerated in patients with oropharyngeal carcinoma

In total, 27 patients with confirmed p16-positive oropharyngeal carcinoma were recruited, as summarized in Supplementary Fig. S3. Data were not included from three patients (one patient had no measurable disease on MRI, one had data acquired with protocol deviations at BL, and one patient had gas inhalation failure at BL). Therefore, 24 patients [median age, 67 years (59–75 IQR), 21 males] were included in the main study for subsequent analysis.

The number of days [mean (± SD)] between double BL imaging sessions was 6 (±2) days. There were 12 (±4) days from radiotherapy start to W2 imaging and 30 (±8) days between radiotherapy start to W4 imaging.

Motion correction was successful in all but four lesion datasets which could not be corrected [patients 11 (tumor) and 20 (tumor), one of two BL scans, patient 13 W4 (tumor and node) scan]. Example motion corrected Δ*R*_1_ time-courses are shown in Supplementary Fig. S4A–S4C. Additionally, contrast agent delivery was not successful in patient 31 at W2, and so data at this timepoint are also not included. The primary tumor of patient 37 had responded to become nonmeasurable by W4 due to excellent clinical response and so it was only possible to include nodal data at this timepoint for this patient. Additional imaging visits are absent at W2 and W4 relating to four patients unable to attend due to COVID-19 infection and isolation or department closure during the pandemic and other patients being unable to attend imaging as they became too unwell, during standard-of-care treatment.

Details of patient demographics, tumor site, stage, treatment regimen, target lesions imaged, and imaging timepoints (i.e., BL0, BL1, W2, and W4), which had useable data, are provided in Table 1. The scan session lasted 50 to 60 minutes, including patient setup. No patient adverse events were reported.

**Table 1.** Clinical information and MRI scan details for image datasets from the 24 patients used in subsequent analysis.

ID	Sex	Age	Disease subsite	TNMv8 stage	Treatment dose (Gy)/fractions, (chemotherapy)	Target lesion	Imaging acquired				MR system
							BL0	BL1	W2	W4	
2	F	74	Tonsil	T3 N1	55/20	T	✔	✔	✔		Diagnostic MR
3	F	56	Tonsil	T4 N0	70/35 and cisplatin (weekly)	T		✔	✔		Diagnostic MR
4	M	72	Tonsil	T2 N1	55/20	T, N	✔	✔	✔	✔	Diagnostic MR
7	M	64	Tonsil	T3 N1	66/30 and carboplatin (weekly)	T, N	✔	✔	✔	✔	Diagnostic MR
9	M	76	Tonsil	T3 N2	55/20	T, N, N		✔	✔		Diagnostic MR
10	M	79	Tonsil	T2 N1	55/20	T, N		✔			Diagnostic MR
11	F	61	Tonsil	T4 N0	66/30 and cisplatin (3-weekly)	T	^a^	✔	✔		Diagnostic MR
12	M	58	Tonsil	T3 N1	66/30 and cisplatin (3-weekly)	T, N, N	✔	✔	✔		Diagnostic MR
13	M	71	Tonsil	T4 N1	70/35 and cisplatin (weekly)	T, N		✔	✔	^a^	Diagnostic MR
14	M	65	Soft palate	T2 N0	66/30	T	✔	✔		✔	MR Linac
16	M	66	Tongue base	T1 N3	66/30 and cisplatin (3-weekly)	T	✔	✔	✔	✔	MR Linac
18	M	60	Tonsil	T3 N1	66/30 and cisplatin (3-weekly)	T, N	✔	✔	✔		MR Linac
19	M	77	Tongue base	T4 N2	66/30	T, N		✔	✔	✔	Diagnostic MR
20	M	67	Tongue base	T2 N2	66/30 and cisplatin (3-weekly)	T, N	✔^a^	✔	✔	✔	Diagnostic MR
21	M	74	Tongue base	T4 N1	66/30	T, N	✔	✔	✔	✔	Diagnostic MR
22	M	67	Tongue base	T2 N3	66/30 and carboplatin (3-weekly)	T, N		✔			Diagnostic MR
23	M	77	Tongue base	T1 N1	55/20	T, N	✔	✔	✔		MR Linac
24	M	53	Tonsil	T4 N1	55/20	T, N		✔			Diagnostic MR
28	M	57	Tonsil	T4 N1	66/30 and cisplatin (3-weekly)	N		✔	✔	✔	Diagnostic MR
29	M	75	Tonsil	T4 N1	55/20	T, N		✔	✔	✔	MR Linac
30	M	75	Tongue base	T1 N1	66/30	N, N		✔			Diagnostic MR
31	M	53	Tonsil	T2 N1	66/30 and cisplatin (3-weekly)	T, N		✔	^b^	✔	MR Linac
35	M	63	Tonsil	T3 N1	66/30 and cisplatin (3-weekly)	T, N	✔	✔	✔	✔	MR Linac
37	M	51	Tongue base	T2 N1	66/30 and cisplatin (3-weekly)	T, N	✔	✔	✔	✔^c^	MR Linac

Target lesions imaged, N, local metastatic lymph node; T, primary tumor.

a Datasets not included in analysis due to motion corruption included patient 11, tumor at BL; patient 13, tumor and node at W4; and patient 20, tumor at BL.

b Contrast agent delivery was not carried out on patient 31 at W2, so data at this timepoint are not included.

c Patient 37 tumor was nonmeasurable at W4 and not included.

BL WTVs are listed for all lesions in Supplementary Table S2. Of the 24 patients (44 lesions), included in the main study, BL HV_MRI_ ranged from 1.3 to 82.1 cm^3^ with a median (IQR) HV_MRI_ of 10.3 cm^3^ (5.7, 24.6).

### OE-MRI biomarkers are repeatable

Repeatability assessment was performed in 12 patients (21 lesions; 11 pimary tumors, 10 nodal lesions; Supplementary Fig. S5A–S5E) who attended BL0 and BL1 imaging sessions. None of the imaging biomarkers were normally distributed and so all underwent log transformation (parameter histograms and Shapiro–Wilk test results provided in Supplementary Fig. S6A–S6E; Supplementary Table S3 respectively). The median values (IQR) of the HV_MRI_ values at the two BL timepoints were 16.0 cm^3^ (6.4, 36.0) and 10.5 cm^3^ (6.6, 43.6). The HV_MRI_ wCV was 24.6%, and the RC LOA were RC_L_ = −45.7% and RC_U_ = 84.1%.

Repeatability information for other imaging biomarkers is listed for comparison in Table 2. Nonperfused volumes of tumor were negligible for all except two patients and are not included further in the analyses.

**Table 2.** Pretreatment repeatability data for 21 lesions from 12 patients acquired at two pretreatment BL timepoints (BL0 and BL1).

Parameter	BL0 [median (IQR)]	BL1 [median (IQR)]	wCV (95% CI)	RC LOA (RC_L_, RC_U_)
Δ*R*_1_ (seconds^−1^)	0.017 (0.011, 0.026)	0.018 (0.013, 0.025)	31.5% (23.5%–48.0%)	−53.2%, 113.8%
HF_MRI_	0.46 (0.30, 0.53)	0.45 (0.29, 0.54)	20.4% (15.3%–30.4%)	−40.2%, 67.2%
HV_MRI_ (cm^3^)	16.0 (6.4, 36.0)	10.5 (6.6, 43.6)	24.6% (18.5%–37.0%)	−45.7%, 84.1%
NV_MRI_ (cm^3^)	21.3 (9.2, 41.9)	20.6 (10.6, 46.2)	16.3% (12.3%–24.0%)	−34.1%, 51.8%
WTV (cm^3^)	37.7 (17.6, 76.7)	35.0 (20.0, 87.2)	10.6% (8.0%–15.4%)	−24.3%, 32.1%

Abbreviations: 95% CI, 95% confidence intervals on wCV.

### HV_MRI_ detects hypoxia modification following therapy across the cohort

Biological response to therapy—here hypoxia modification—was examined in the cohort by assessing the change in HV_MRI_ from pretreatment BL to W2 and W4. BL was defined as either BL1, for those with one BL visit, or as the mean of BL measurements, for those with two BL visits (i.e., BL0 and BL1). In all, 20 patients (36 lesions: 19 primary tumors; 17 nodal lesions), with a BL scan and at least a W2 or W4 scan or both, were evaluated.

**Figure 1.**
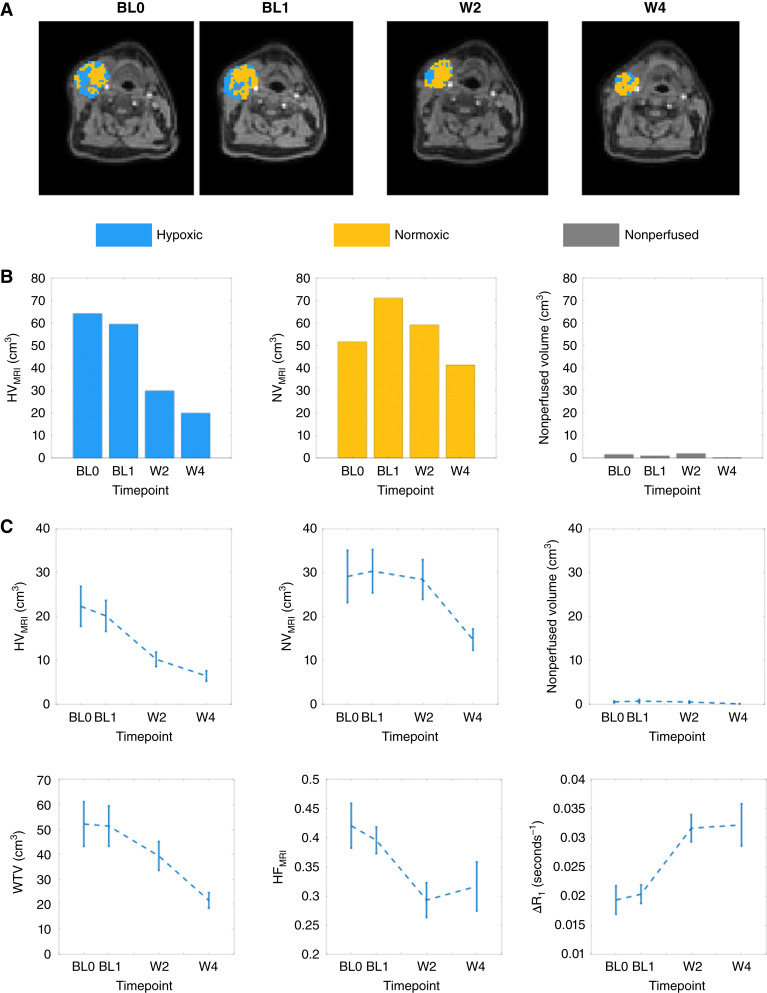
Treatment-induced changes in OE-MRI–derived biomarkers. **A,** Example hypoxia maps for a large metastatic lymph node (patient 7) obtained at two BL timepoints (BL0 and BL1) and W2 and W4 of radiation treatment. Corresponding Δ*R*_1_ maps are provided in Supplementary Fig. S7A–S7C. **B,** Bar charts plot the relative sizes of HV_MRI_ along with NV_MRI_ and nonperfused volume for this patient. Note: The combination of HV_MRI_, NV_MRI_, and nonperfused volume equate to the WTV. **C,** Patient cohort assessment of treatment effects illustrated as plots of standard error of the mean (SEM) for (top left to bottom right) HV_MRI_, NV_MRI_, nonperfused volume, WTV, HF_MRI_, and Δ*R*_1_.

Example Δ*R*_1_ data (Supplementary Fig. S7A–S7C) and hypoxia maps are provided (Fig. 1A) for the nodal tumor in patient 7. In this example, HV_MRI_ shows reduction beyond the RC LOA threshold during treatment (i.e., reduction beyond RC_L_ = −45.7% from BL). Results for the comparative lack of change in NV_MRI_ and nonperfused tissue are shown (Fig. 1B).

At W2, 18 patients (33 lesions: 17 primary tumors; 16 nodal lesions) had imaging. A cohort-level reduction in HV_MRI_ was observed, with a median BL hypoxic volume of 11.3 cm^3^ (6.8, 28.3) that reduced at W2 to 6.9 cm^3^ (3.5, 13.0; *P* < 0.001; Supplementary Table S4). At W4, imaging was only obtained in 12 patients, but a significant cohort-level reduction in HV_MRI_ was detected (20 lesions: 10 primary tumors, 10 nodal lesions) from BL down to 5.9 cm^3^ (2.4, 8.6; *P* < 0.001). The corresponding mean (± SE) values of HV_MRI_ are 20.2 (± 3.5) cm^3^ (BL), 10.3 (± 1.7) cm^3^ (W2), and 6.5 (± 1.2) cm^3^ (W4).

For comparison, a significant increase in Δ*R*_1_ and decrease in HF_MRI_ were observed at W2 and W4 (*P* ≤ 0.001; Supplementary Table S4). In distinction, NV_MRI_ did not change at W2, whereas the overall WTV was reduced (*P* < 0.001). Cohort changes for all imaging biomarkers are displayed as plots of the standard error of the mean (SEM) in Fig. 1C, and changes in individual lesions are also provided (Supplementary Fig. S8A–S8E).

### HV_MRI_ identifies the incidence and onset of hypoxia modification

The change in HV_MRI_ was calculated for each lesion. This was compared with the RC LOA, (%RC_L_, %RC_U_ = −45.7%, 84.1%; see Table 2) to determine if HV_MRI_ increased or decreased in an individual lesion more than could be expected by chance (30).

**Figure 2.**
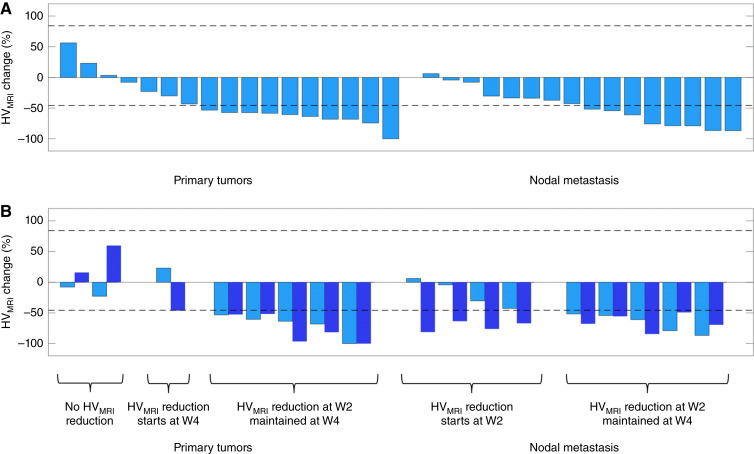
HV_MRI_ changes at W2. **A,** Waterfall plot showing the percentage change in HV_MRI_ from BL to W2 in all lesions, categorized as primary tumors or nodal metastases. **B,** Analysis of the 17 lesions imaged at both W2 and W4 compares the reduction from BL at W2 (light blue) and W4 (dark blue). Dashed lines are the asymmetrical RC LOA for HV_MRI_ (i.e., −45.7% and +84.1%).

Hypoxia modification was identified. Data were assessed in 18 patients (with 33 lesions) at W2 and in 12 patients (20 lesions) at W4. At W2, 18/33 (54.5%) of all lesions had a significant reduction in HV_MRI_. Overall, 10/17 (58.8%) primary tumors and 8/16 (50.0%) nodal lesions had lesion-specific reduction in HV_MRI_ (Fig. 2A). At W4, there was even greater evidence of hypoxia change, with a significant reduction in HV_MRI_ in 15/17 (88.2%) of all lesions, comprising 6/8 (75.0%) primary tumors and 9/9 (100%) nodal lesions. In all, 36 lesions were evaluated at W2 and/or W4. Of these, 26/36 (72.2%) showed reduction in HV_MRI_ by their last scan.

The persistence of hypoxia modification was examined in 10 patients (comprising 17 lesions) who had scans at both W2 and W4. Of these, 2/17 (11.8%) lesions had no reduction in HV_MRI_ at W2 or W4; 5/17 (29.4%) lesions had reduction in HV_MRI_ that was only significant by W4; 10/17 (58.8%) lesions had reduction in HV_MRI_ by W2, all of which showed persistent hypoxia modification at W4 (Fig. 2B).

### HV_MRI_ characterizes concordance in hypoxia modification in primary and nodal tumors

Changes in HV_MRI_ were examined at W2 or W4 for the 14 patients who had both primary and nodal lesions. The presence or absence of HV_MRI_ change beyond the RC LOA limits was recorded. Fully concordant change—in which both primary and nodal lesions behaved in the same manner at all available image timepoints—was seen in 7/14 (50.0%) patients.

Notably, fully discordant changes occurred in 6/14 patients (42.9%), which was marked in four patients (Table 3). This included examples both of primary tumors having hypoxia modification whereas nodal tumors remained unchanged (patients 7 and 20) and, conversely, of primary tumors remaining unchanged whereas nodal tumors showed hypoxia modification (patients 9 and 37). In two of the cases, discordant changes were more marginal (patients 12 and 18). Finally, one patient (patient 29) had initial discordant change in their primary and nodal lesions at W2, followed by concordant change (reduction in HV_MRI_) at W4.

**Table 3.** Evaluation of concordance of hypoxia modification in patients with two or more lesions, by assessing serial values of HV_MRI_ at pretreatment and W2 and W4.

Patient ID	Lesion	HV_MRI_ W2	HV_MRI_ W4	Concordant change between lesions?	Comment
4	T	**↓**	**↓**	Yes	
	N	**↓**	**↓**		
7	T	NC	NC	No	N had reduced HV_MRI_ at both W2 and W4, but T did not
	N	**↓**	**↓**		
9	T	**↓**		No	T had reduced HV_MRI_ at W2, but N1 and N2 did not
	N1	NC			
	N2	NC			
12	T	**↓**		No (but equivocal)	T and N2 reduced HV_MRI_ at W2; N1 hypoxia decrease of 37.1% approached the RC of −45.7%
	N1	NC (↓)			
	N2	**↓**			
13	T	**↓**		Yes	
	N	**↓**			
18	T	NC (↓)		No (but equivocal)	N reduced HV_MRI_ at W2; T hypoxia decrease of 43.1% approached the RC of −45.7%
	N	↓			
19	T	NC	**↓**	Yes	
	N	NC	**↓**		
20	T	NC	NC	No	T hypoxia increased 59.7% at W4, whereas N reduced HV_MRI_; neither lesion changed at W2
	N	NC	**↓**		
21	T	**↓**	**↓**	Yes	
	N	**↓**	**↓**		
23	T	NC		Yes	
	N	NC			
29	T	**↓**	**↓**	No (at W2); yes (at W4)	Concordance does not manifest until W4
	N	NC	**↓**		
31	T		**↓**	Yes	
	N		**↓**		
35	T	**↓**	**↓**	Yes	
	N	**↓**	**↓**		
37	T	**↓**		No	T had reduced HV_MRI_ at W2, but N did not
	N	NC	**↓**		

If HV_MRI_ was reduced beyond the RC of −45.7%, then HV_MRI_ for that lesion is reduced (↓); otherwise, there is no change (NC) measured.

### HV_MRI_ and WTV reduction have multiple distinct patterns

**Figure 3.**
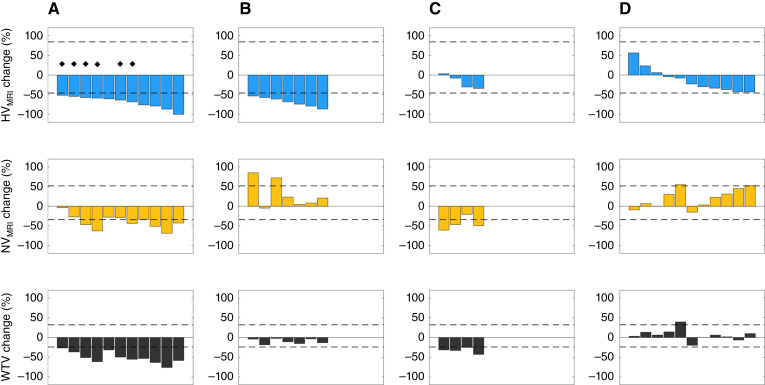
Waterfall plots showing change in HV_MRI_, NV_MRI_, and WTV at W2. Four patterns seen were (**A**) lesions with significant hypoxia modification and reduction in WTV. Diamonds indicate tumors in which the hypoxic fraction remained unchanged; **B,** tumors with significant hypoxia modification that did not have change in WTV; (**C**) tumors that did not have significant individual changes in hypoxia, but significant reduction in WTV was driven by reduction in NV_MRI_; (**D**) tumors with no reduction in HV_MRI_, NV_MRI_, or WTV.

The change in HV_MRI_ at W2 was compared with changes in normoxic volume (NV_MRI_) and WTV. At W2, the 18/33 lesions with hypoxia modification had two distinct patterns of change. Eleven lesions had significant reduction in NV_MRI_ and therefore had associated reduction in overall WTV (Fig. 3A). In distinction, seven lesions had no significant change in WTV despite reduction in HV_MRI_ because NV_MRI_ did not reduce significantly and in some cases increased beyond the RC LOA (Fig. 3B).

The remaining 15/33 lesions had no hypoxia modification at W2 (i.e., HV_MRI_ did not reduce beyond the RC LOA). Of these, four lesions had significant reduction in WTV despite lack of hypoxia modification, driven by significant reduction in NV_MRI_ (Fig. 3C). Eleven lesions had no reduction in WTV relating to no significant reduction in either HV_MRI_ or NV_MRI_ (indeed, one lesion had a significant increase in WTV, driven by an increase in NV_MRI_; Fig. 3D).

We also compared the hypoxia change induced by therapy by measuring HF_MRI_ and HV_MRI_. Changes in HF_MRI_ mirrored changes in HV_MRI_ except for six lesions in which HF_MRI_ did not change despite reduction in HV_MRI_. In these six lesions, WTV, HV_MRI_, and NV_MRI_ all reduced in similar proportions, thereby not affecting the proportion of hypoxic to normoxic tissue and rendering HF_MRI_ insensitive to radiation-induced treatment effects.

## Discussion

Hypoxia is a major driver of resistance to therapy in patients with HNSCC including those with HPV-associated oropharyngeal carcinoma (2). Data from hypoxia PET, the most common modality used to image hypoxia (32), suggest that persistent low tumor oxygenation during early treatment with radiotherapy predicts treatment failure (11, 12). Prospective trials of adaptive radiotherapy based on early change in PET hypoxia status, for example when identified from FMISO-PET imaging, suggest a potential strategy to personalize dose delivery and improve disease control while reducing unnecessary radiotherapy-related side effects (9, 33). In particular, recent data from the 30 ROC trials suggest that hypoxia measurement is important for the management of dose de-escalation strategies (8).

Sparse hypoxia tracer availability, high cost, and limited infrastructure capable of performing PET hypoxia imaging have hindered widespread clinical adoption. OE-MRI is an affordable and practical alternative to PET for the assessment of radiation-induced hypoxia modification in patients with non–small cell lung cancer (NSCLC) (23). Here, we sought to determine if OE-MRI HV_MRI_ was repeatable in patients with newly diagnosed p16-positive oropharyngeal carcinoma. We then aimed to evaluate the incidence, onset, and variation in hypoxia modification induced by (chemo)radiotherapy.

Our data provide evidence that OE-MRI, when combined with perfusion imaging, is feasible, tolerable, and can detect cohort changes in hypoxia, thus offering an alternative to hypoxia PET imaging in patients with HNSCC. Oxygen enhancement in tissues was measured by Δ*R*_1_, which indicated technique success in all patients, consistent with an independent study (34) but contrary to findings in HNSCC from a different laboratory (35). Δ*R*_1_ increase observed in our patient population is consistent with tissue re-oxygenation (36) following fractionated radiotherapy. Median values and ranges of Δ*R*_1_, HV_MRI_, and HF_MRI_ were defined. Cohort-level reductions were similar to those reported with OE-MRI HV_MRI_ in NSCLC (23) and in hypoxic volume using FMISO-PET (11, 12).

A key feature of this study was our examination of measurement precision. HV_MRI_ showed good repeatability with a wCV of 24.6%, comparable with previous data in NSCLC (in which HV_MRI_ wCV = 25.9%; ref. 23). Other OE-MRI parameters and measures of WTV also showed good repeatability that are comparable with other quantitative imaging biomarkers such as *K*^trans^ (37–39). The RC LOA determined if and when therapy-induced changes in the HV_MRI_ of individual lesions could be considered real at a 95% confidence level (26). Identifying those lesions which experienced significant changes in hypoxia and those which did not enabled three key findings to be noted, each with translational implications.

First, we identified the variable onset of hypoxia modification in individual lesions. We showed that 54.5% of all lesions had reduced HV_MRI_ at W2, with similar proportions of primary tumors and nodal metastases changing. The proportion of lesions exhibiting hypoxia reduction increased to nearly 90% at W4, with the caveat that fewer lesions were examined at that timepoint. Notably, all lesions with hypoxia reduction by W2 had persistent change to W4 and of equal note, some lesions with significant hypoxia modification only manifested this change by W4. This information provides insight beyond that derived from cohort analysis alone (40) and implies that dose de-escalation may be performed in around half of patients within 2 weeks of radiation-based therapy to achieve maximal benefit. In addition, more moderate dose de-escalation could be of benefit to another group of patients who show detectable hypoxia modification between W2 to W4 of radiotherapy, although larger studies will need to determine the optimum patient benefit and cost-effectiveness.

Second, we examined patients with both primary tumors and nodal metastases. It is known that patients can have varying levels of hypoxia in their primary and nodal HNSCC tumors (15). However, the relative incidence of concordance and discordance of lesion hypoxia between primary and nodal tumors is not well understood. We identified that only half of patients had concordant hypoxia modification in both lesions, and that primary tumor and nodal metastases behaved differently in the other half of patients (lesion changes were discordant). This implies that sampling only a single lesion through imaging or biopsy may provide an inaccurate picture of the change in hypoxic status in a substantial proportion of clinical cases. Clinical decisions—such as whether to dose de-escalate or not—require an assessment of all locoregional lesions rather than one index lesion and may require individual dosing to specific nodal levels. It is even possible that future strategies may allow de-escalated treatment in nodes that demonstrate biological response while maintaining dose in others that do not have hypoxia modification. Such approaches become possible in the era of functional imaging assessment performed at regular on-therapy intervals on systems such as an MR Linac (41).

Third, we showed that hypoxic volumes reduced significantly in more than 20% of lesions that did not demonstrate overall size reduction in the early response to therapy. This implies that a number of patients have biological changes in their lesions that are not detected through conventional assessment of tumor size. Further work in larger patient numbers is needed to assess the relative importance of hypoxia modification as an additional covariate in predicting the clinical outcome. In addition, we highlight the potential limitations of solely measuring hypoxic fraction (HF_MRI_) as this measure can seem insensitive to treatment effects when both the hypoxic subvolume and the overall tumor volume are reduced in an approximately equivalent ratio (42).

Some study limitations existed. Although this study is the largest clinical OE-MRI study performed to date, several patients had missing data due to factors including scan cancellations during the COVID-19 pandemic. It should also be noted that, in line with the general HNSCC population, patients recruited to this study were p16-positive, which is considered an accepted method of identifying HPV-associated disease, and these patients are known to respond superiorly to those with p16-negative HNSCC (43). Further work should assess whether similar OE-MRI data are obtained in patients with p16-negative disease.

Future studies are required to extend biological validation already obtained from multiple animal models (18–21), with hypoxia gene signatures and other methods in appropriate HNSCC clinical population. In addition, establishment of multicenter reproducibility in larger numbers of patients, across multiple vendor platforms and field strength, will provide a more definitive estimate of RC LOA for use in further clinical studies. Refinement of estimates of HV_MRI_ may also be required to distinguish acute transient hypoxia from established chronic hypoxia, the former of which may contribute to part of the variation in biomarker estimates between the two BL scans.

Collectively, the data reported here add to our previous work in NSCLC and HNSCC (23, 24) to support the use of OE-MRI as a biological response assay. The technique can identify the onset, persistence, and variation in hypoxia modification in different lesions and different patients, making a strong case for the value of imaging assessment in studies that evaluate tumor hypoxia.

## Supplementary Material

Supplementary Figure S1 Supplementary Figure S1. Summary of MR imaging protocol and gas challenge timings.

Supplementary Figure S2 Supplementary Figure S2. Illustration of the data that is used to derive the ΔR1, HFMRI and HVMRI parameters.

Supplementary Figure S3 Supplementary Figure S3. Patient recruitment and imaging sample size.

Supplementary Figure S4 Supplementary Figure S4. Motion correction effect on ΔR1 time-series.

Supplementary Figure S5 Supplementary Figure S5. OE-MRI biomarker repeatability.

Supplementary Figure S6 Supplementary Figure S6. Histograms of baseline OE-MRI biomarker results.

Supplementary Figure S7 Supplementary Figure S7. Treatment-induced changes in OE-MRI biomarker ΔR1.

Supplementary Figure S8 Supplementary Figure S8. Treatment-induced changes in OE-MRI biomarkers relative to baseline (BL).

Supplementary Table S1 Supplementary Table S1. MR sequence parameters for T1 mapping, OE-MRI and DCE-MRI sequences.

Supplementary Table S2 Supplementary Table S2. Whole tumor volume (WTV) measurements for each lesion (T = Primary Tumor, N = Metastatic Lymph Node) and patient at the baseline (BL).

Supplementary Table S3 Supplementary Table S3. Results from repeatability assessment including results from Shapiro-Wilks test for normality and whether data were subsequently log-transformed to obtain wCV and RC data.

Supplementary Table S4 Supplementary Table S4. Summary of cohort lesion parameter median values at baseline and W2 and W4.
