# Supplementary material for: Oxygen-Enhanced MRI Detects Incidence, Onset, and Heterogeneity of Radiation-Induced Hypoxia Modification in HPV-Associated Oropharyngeal Cancer
Source: Clin Cancer Res. 2024 Aug 9;30(24):5620–9. doi: 10.1158/1078-0432.CCR-24-1170 (PMC11654720; doi:10.1158/1078-0432.CCR-24-1170)
Supplement: Supplementary Figure S1 — Summary of MR imaging protocol and gas challenge timings. [file ccr-24-1170_supplementary_figure_s1_suppsf1.docx]

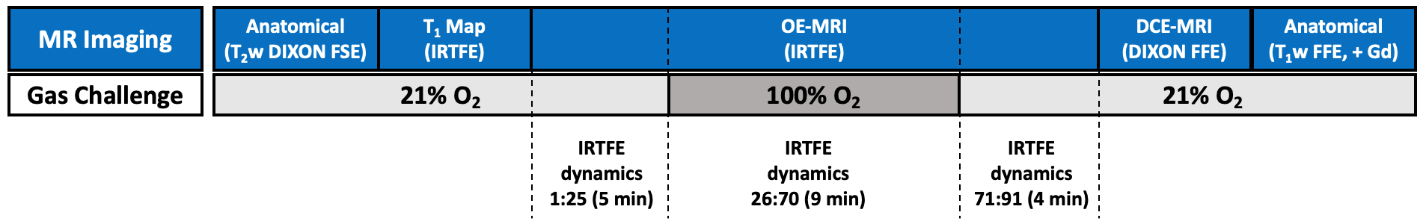


**Supplementary Figure S1**. Summary of MR imaging protocol and gas challenge timings. Sequences include: FSE = Fast Spin Echo, IRTFE = Inversion-Recovery Turbo Field Echo, FFE = Fast Field Echo, Gd = Gadolinium. OE-MRI = Oxygen Enhanced MRI, DCE-MRI = Dynamic Contrast Enhanced MRI.
