# Supplementary material for: Oxygen-Enhanced MRI Detects Incidence, Onset, and Heterogeneity of Radiation-Induced Hypoxia Modification in HPV-Associated Oropharyngeal Cancer
Source: Clin Cancer Res. 2024 Aug 9;30(24):5620–9. doi: 10.1158/1078-0432.CCR-24-1170 (PMC11654720; doi:10.1158/1078-0432.CCR-24-1170)
Supplement: Supplementary Figure S2 — Illustration of the data that is used to derive the ΔR1, HFMRI and HVMRI parameters. [file ccr-24-1170_supplementary_figure_s2_suppsf2.docx]

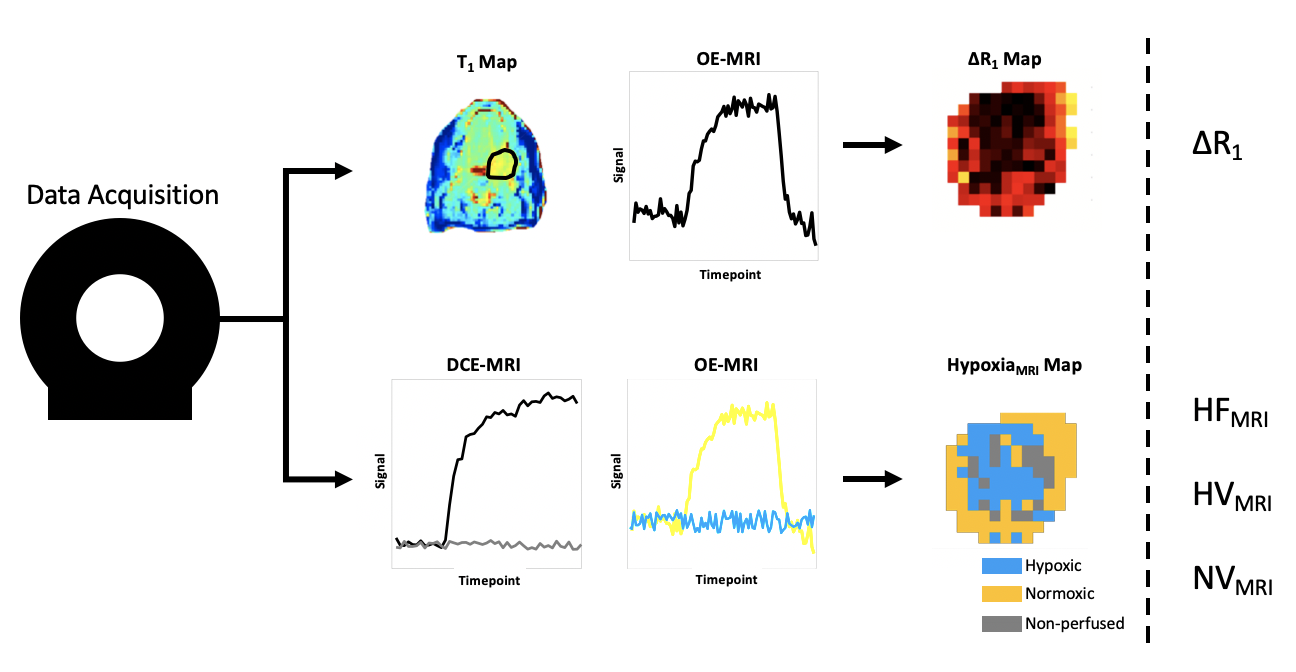


**Supplementary Figure S2**. Illustration of the data that is used to derive the ΔR_1_, HF_MRI_ and HV_MRI_ parameters. Following data acquisition, motion correction and registration; (Top) the OE-MRI signal time course is combined with the T_1_ map (T_1_ map of whole neck shown with tumour region overlaid in black) to produce maps of ΔR_1_ from which the median ΔR_1_ can be derived. (Bottom) The DCE-MRI signal time course allows voxels to be categorized as ‘gadolinium enhancing’, which are labelled perfused, or ‘gadolinium non-enhancing’, labelled non-perfused. The OE-MRI signal time course per-voxel allows voxels to be categorized as ‘oxygen enhancing’ or ‘non-oxygen-enhancing’. Voxels which are ‘perfused’ and ‘oxygen enhancing’ are labelled ‘Normoxic’ and those which are ‘perfused’ but ‘non-oxygen-enhancing’ are labelled ‘Hypoxic’. The fraction of the lesion which is ‘Hypoxic’ is then termed the MRI hypoxic fraction (HF_MRI_) and similarly the ‘Hypoxic’ volume is labelled HV_MRI_ and the ‘Normoxic’ volume is labelled as NV_MRI_.
