# Supplementary material for: Oxygen-Enhanced MRI Detects Incidence, Onset, and Heterogeneity of Radiation-Induced Hypoxia Modification in HPV-Associated Oropharyngeal Cancer
Source: Clin Cancer Res. 2024 Aug 9;30(24):5620–9. doi: 10.1158/1078-0432.CCR-24-1170 (PMC11654720; doi:10.1158/1078-0432.CCR-24-1170)
Supplement: Supplementary Figure S3 — Patient recruitment and imaging sample size. [file ccr-24-1170_supplementary_figure_s3_suppsf3.docx]

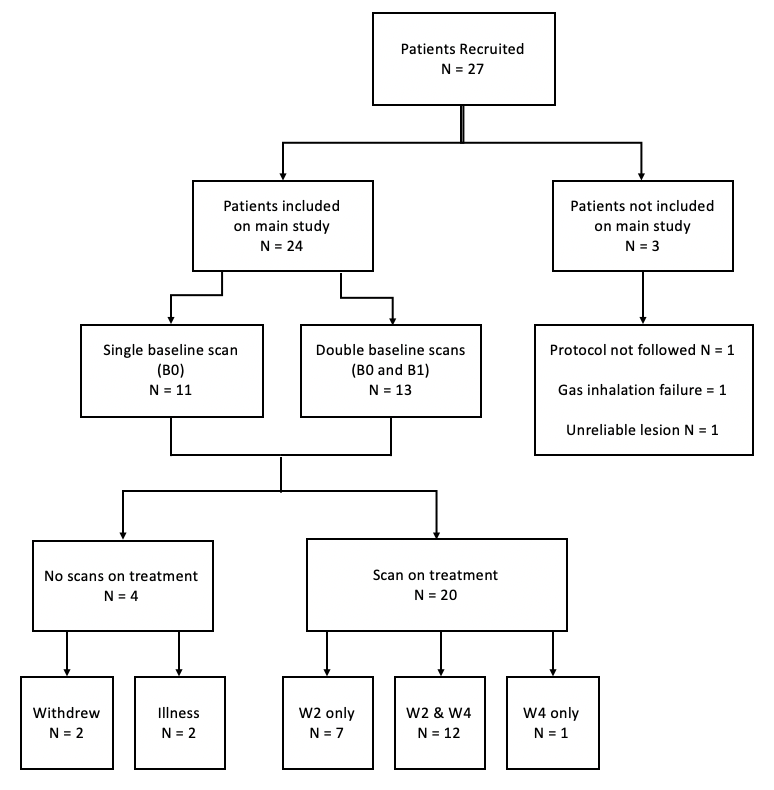


**Supplementary Figure S3**. Patient recruitment and imaging sample size. Flow diagram depicting the number of patients that were included in the study and how many patients attended each of the imaging scan sessions, as well as numbers of patients who left the study.
