# Supplementary material for: Oxygen-Enhanced MRI Detects Incidence, Onset, and Heterogeneity of Radiation-Induced Hypoxia Modification in HPV-Associated Oropharyngeal Cancer
Source: Clin Cancer Res. 2024 Aug 9;30(24):5620–9. doi: 10.1158/1078-0432.CCR-24-1170 (PMC11654720; doi:10.1158/1078-0432.CCR-24-1170)
Supplement: Supplementary Figure S4 — Motion correction effect on ΔR1 time-series. [file ccr-24-1170_supplementary_figure_s4_suppsf4.docx]

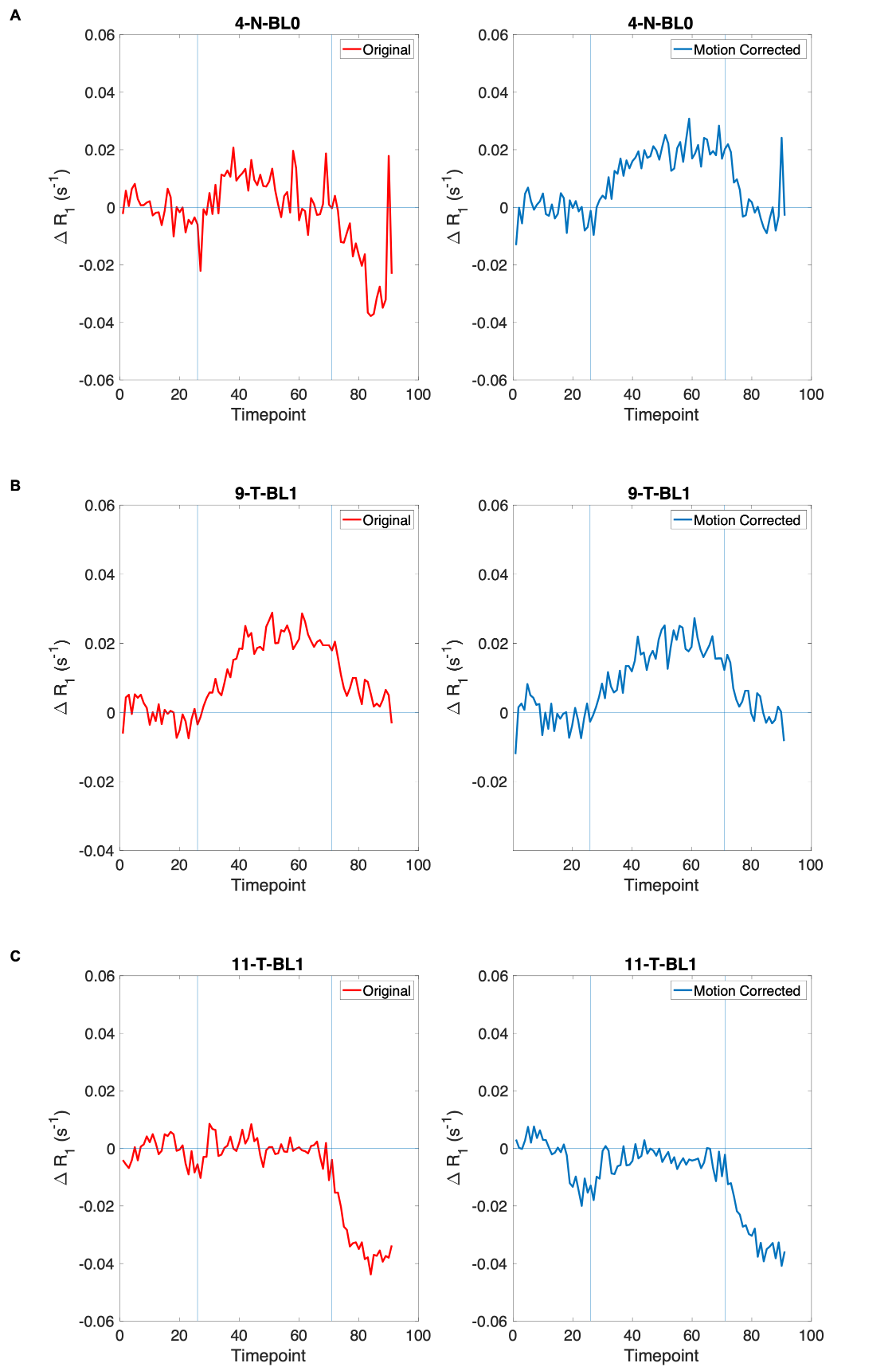


**Supplementary Figure S4**. Motion correction effect on ΔR_1_ time-series. Example ΔR_1_ time-series from uncorrected data (left) and following motion-correction (right) of the dynamic OE-MRI IRTFE sequence, showing: (A) satisfactory motion-correction for a lymph node of patient 4, baseline visit BL0 (i.e. 4-N-BL0), (B) satisfactory motion-correction for 9-T-BL1 without causing unnecessary registration when not required, and (C) unresolvable motion artefact following motion correction with 11-T-BL1, as such this dataset was removed from further analysis in the study. Reference lines at timepoints t = 26 and t = 71 represent timepoints of gas switching from air-100% O_2_ and 100% O_2_-air respectively.
