# Supplementary material for: Oxygen-Enhanced MRI Detects Incidence, Onset, and Heterogeneity of Radiation-Induced Hypoxia Modification in HPV-Associated Oropharyngeal Cancer
Source: Clin Cancer Res. 2024 Aug 9;30(24):5620–9. doi: 10.1158/1078-0432.CCR-24-1170 (PMC11654720; doi:10.1158/1078-0432.CCR-24-1170)
Supplement: Supplementary Figure S5 — OE-MRI biomarker repeatability. [file ccr-24-1170_supplementary_figure_s5_suppsf5.docx]

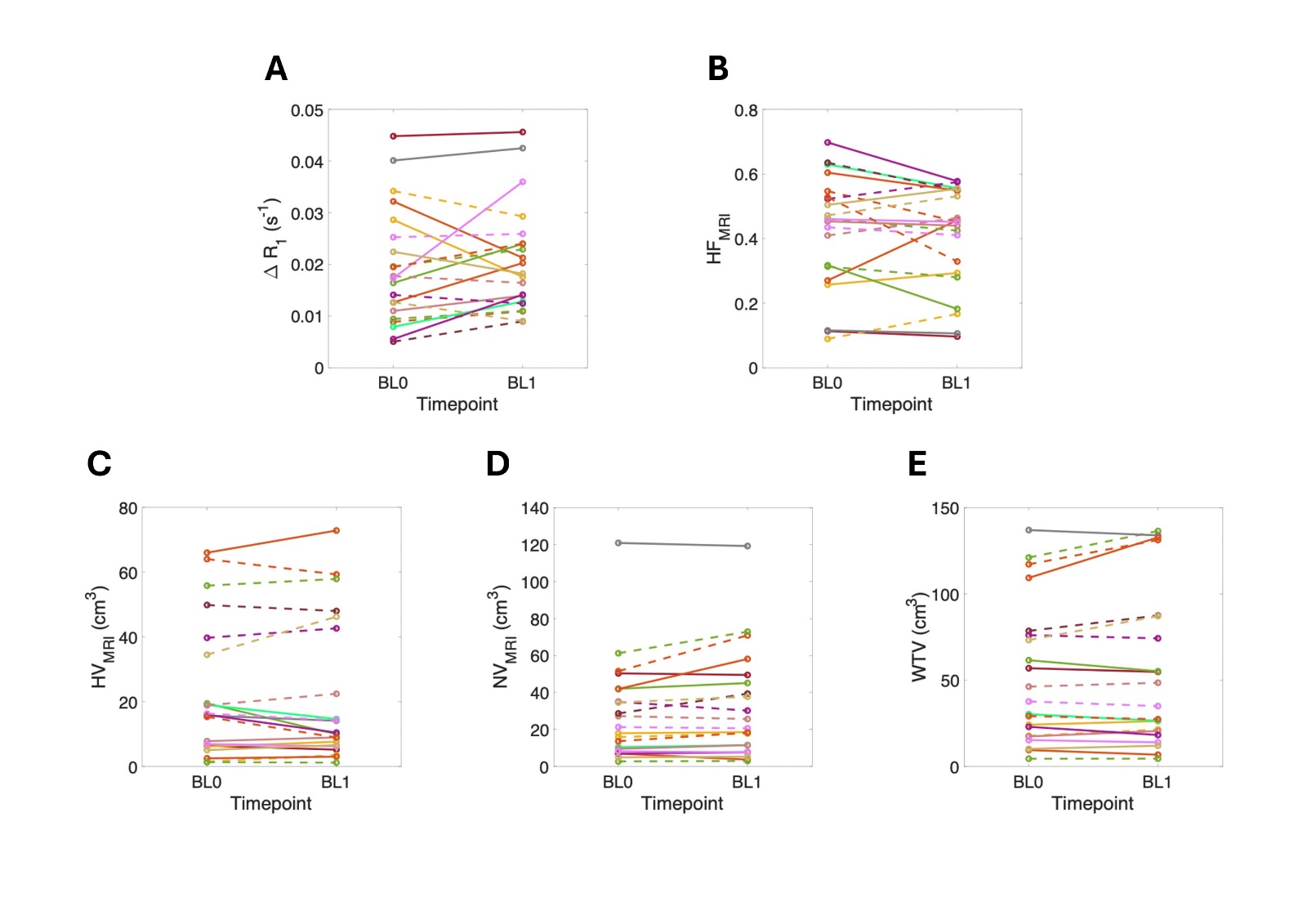


**Supplementary Figure S5**. OE-MRI biomarker repeatability. Line plots showing the baseline parameter values (BL0 and BL1) for imaging biomarkers: (A) ΔR_1_, (B) HF_MRI_, (C) HV_MRI_, (D) NV_MRI_, and (E) WTV. Solid lines = primary tumor, dashed lines = involved lymph nodes, and each colour represents a different patient.
