# Supplementary material for: Oxygen-Enhanced MRI Detects Incidence, Onset, and Heterogeneity of Radiation-Induced Hypoxia Modification in HPV-Associated Oropharyngeal Cancer
Source: Clin Cancer Res. 2024 Aug 9;30(24):5620–9. doi: 10.1158/1078-0432.CCR-24-1170 (PMC11654720; doi:10.1158/1078-0432.CCR-24-1170)
Supplement: Supplementary Figure S6 — Histograms of baseline OE-MRI biomarker results. [file ccr-24-1170_supplementary_figure_s6_suppsf6.docx]

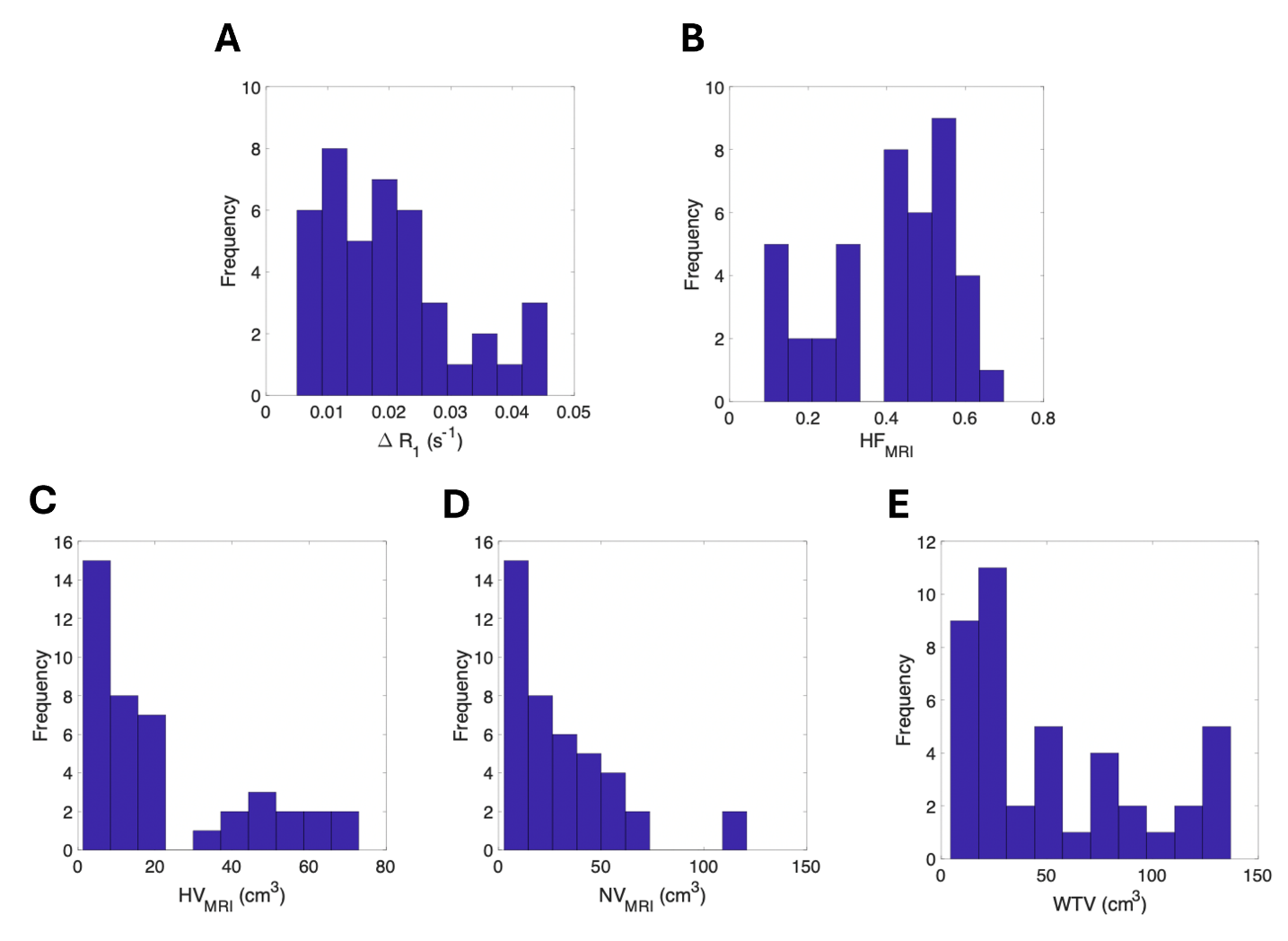


**Supplementary Figure S6**. Histograms of baseline OE-MRI biomarker results. Histograms showing the distributions of the baseline parameter values which were used in repeatability analysis; (A) ΔR_1_, (B) HF_MRI_, (C) HV_MRI_, (D) NV_MRI_, and (E) WTV.
