# Supplementary material for: Oxygen-Enhanced MRI Detects Incidence, Onset, and Heterogeneity of Radiation-Induced Hypoxia Modification in HPV-Associated Oropharyngeal Cancer
Source: Clin Cancer Res. 2024 Aug 9;30(24):5620–9. doi: 10.1158/1078-0432.CCR-24-1170 (PMC11654720; doi:10.1158/1078-0432.CCR-24-1170)
Supplement: Supplementary Figure S7 — Treatment-induced changes in OE-MRI biomarker ΔR1. [file ccr-24-1170_supplementary_figure_s7_suppsf7.docx]

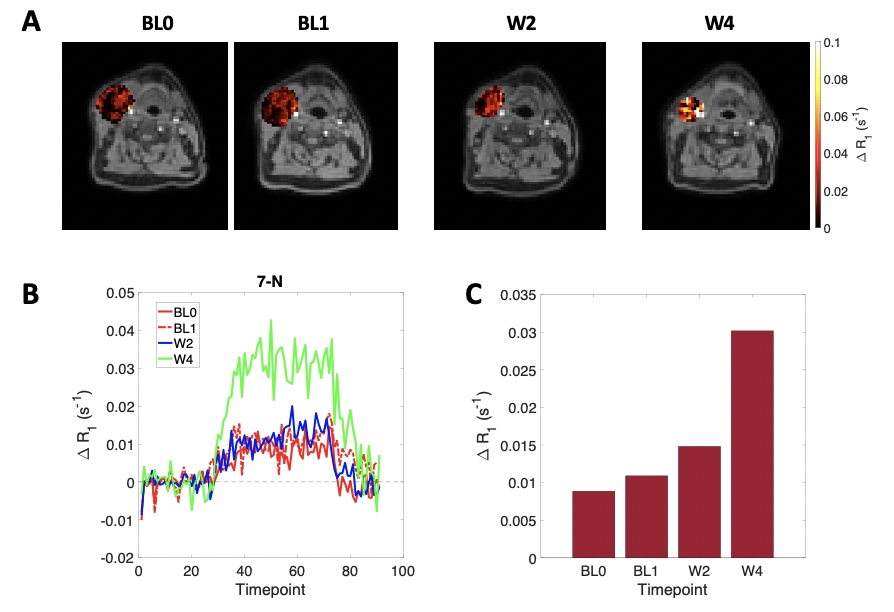


**Supplementary Figure S7**. Treatment-induced changes in OE-MRI biomarker ΔR_1_. (A) Example ΔR_1_ maps for a large metastatic lymph node (Patient 7) obtained at two baseline timepoints (BL0, BL1) and weeks 2 (W4) and 4 (W4) of radiotherapy treatment. Corresponding hypoxia maps are provided in Figure 2 of main text. (B) ΔR_1_ traces obtained from the four visits and (C) bar charts showing median ΔR_1_ values measured at each visit.
