# Supplementary material for: Oxygen-Enhanced MRI Detects Incidence, Onset, and Heterogeneity of Radiation-Induced Hypoxia Modification in HPV-Associated Oropharyngeal Cancer
Source: Clin Cancer Res. 2024 Aug 9;30(24):5620–9. doi: 10.1158/1078-0432.CCR-24-1170 (PMC11654720; doi:10.1158/1078-0432.CCR-24-1170)
Supplement: Supplementary Table S1 — MR sequence parameters for T1 mapping, OE-MRI and DCE-MRI sequences. [file ccr-24-1170_supplementary_table_s1_suppst1.docx]

| **Sequence Parameters** | **Diagnostic MR** | **MR Linac** |
| --- | --- | --- |
| **MR System** | Philips Ingenia (1.5 T) | Elekta-Philips Unity (1.5 T) |
| **Receive Coils** | 16 channel spine array  32 channel large anterior flex (on coil bridge)  2 x 1 channel loop coil (either side of head) | 4 channel posterior coil  4 channel anterior coil (on coil bridge) |
| **T_1_ Mapping** |  | |
| **Sequence** | 3D IRTFE | |
| **TR/TE** | 2.8/0.9 ms | 3.0/1.0 ms |
| **TI** | 100, 500, 800, 1100, 4300 ms | |
| **⍺** | 6° | |
| **OE-MRI** |  | |
| **Sequence** | 3D IRTFE | |
| **TR/TE** | 2.8/0.9 ms | 3.0/1.0 ms |
| **TI** | 1100 ms | |
| **⍺** | 6° | |
| **Dynamic Measurements** | 91 | |
| **Temporal Resolution** | 12 s | |
| **Gas Challenge Protocol** | 5:9:4 mins (21% O_2_ : 100% O_2_ : 21% O_2_)  15 L/min high-concentration mask | |
| **DCE-MRI** |  | |
| **Sequence** | 3D mDIXON FFE | |
| **TR/TE_1_/TE_2_** | 3.2/1.2/2.0 ms | 3.9/1.6/2.6 ms |
| **⍺** | 5° | |
| **Dynamic Measurements** | 45 | |
| **Temporal Resolution** | 3.8 s | 4.2 s |
| **Contrast Protocol** | DOTAREM, 0.2ml/kg, 3ml/s (injection image = 8) | |
| **All Sequences** |  | |
| **Matrix / Slices** | 128 x 128 pixels / 45 Slices | |
| **Resolution** | 3 x 3 x 5 mm^3^ | |

**Supplementary Table S1**. MR sequence parameters for T_1_ mapping, OE-MRI and DCE-MRI sequences. IRTFE = Inversion-Recovery Turbo Field Echo, FFE = Fast Field Echo. TR = Repetition Time, TE = Echo Time, TI = Inversion Delay Time, ⍺ = Flip Angle. All imaging was acquired in the transverse plane.
