# Supplementary material for: Oxygen-Enhanced MRI Detects Incidence, Onset, and Heterogeneity of Radiation-Induced Hypoxia Modification in HPV-Associated Oropharyngeal Cancer
Source: Clin Cancer Res. 2024 Aug 9;30(24):5620–9. doi: 10.1158/1078-0432.CCR-24-1170 (PMC11654720; doi:10.1158/1078-0432.CCR-24-1170)
Supplement: Supplementary Table S2 — Whole tumor volume (WTV) measurements for each lesion (T = Primary Tumor, N = Metastatic Lymph Node) and patient at the baseline (BL). [file ccr-24-1170_supplementary_table_s2_suppst2.docx]

| ID | Lesion | Baseline WTV (cm^3^) |
| --- | --- | --- |
| 2 | T | 55.9 |
| 3 | T | 15.5 |
| 4 | T | 25.2 |
| 4 | N | 19.6 |
| 7 | T | 8.1 |
| 7 | N | 124.2 |
| 9 | T | 34.4 |
| 9 | N1 | 32.3 |
| 9 | N2 | 7.0 |
| 10 | T | 2.5 |
| 10 | N | 2.1 |
| 11 | T | 46.0 |
| 12 | T | 58.4 |
| 12 | N1 | 128.8 |
| 12 | N2 | 4.5 |
| 13 | T | 61.7 |
| 13 | N | 221.1 |
| 14 | T | 28.4 |
| 16 | T | 135.5 |
| 18 | N | 47.4 |
| 18 | T | 19.0 |
| 19 | T | 24.8 |
| 19 | N | 23.4 |
| 20 | T | 15.3 |
| 20 | N | 83.0 |
| 21 | T | 20.6 |
| 21 | N | 75.2 |
| 22 | T | 4.9 |
| 22 | N | 15.7 |
| 23 | T | 14.6 |
| 23 | N | 36.3 |
| 28 | N | 65.3 |
| 29 | T | 91.6 |
| 29 | N | 21.0 |
| 30 | N1 | 1.9 |
| 30 | N2 | 2.0 |
| 31 | T | 26.7 |
| 31 | N | 8.7 |
| 35 | N | 28.3 |
| 35 | T | 121.1 |
| 37 | N | 80.2 |
| 37 | T | 11.1 |

**Supplementary Table S2**. Whole tumor volume (WTV) measurements for each lesion (T = Primary Tumor, N = Metastatic Lymph Node) and patient at the baseline (BL). BL is defined as either BL1, for those with one baseline visit, or as the mean of baseline measurements, for those with two baseline visits (i.e. BL0 and BL1).
