# Supplementary material for: Oxygen-Enhanced MRI Detects Incidence, Onset, and Heterogeneity of Radiation-Induced Hypoxia Modification in HPV-Associated Oropharyngeal Cancer
Source: Clin Cancer Res. 2024 Aug 9;30(24):5620–9. doi: 10.1158/1078-0432.CCR-24-1170 (PMC11654720; doi:10.1158/1078-0432.CCR-24-1170)
Supplement: Supplementary Table S3 — Results from repeatability assessment including results from Shapiro-Wilks test for normality and whether data were subsequently log-transformed to obtain wCV and RC data. [file ccr-24-1170_supplementary_table_s3_suppst3.docx]

| **Parameter** | **ΔR_1_** | **HF_MRI_** | **HV_MRI_** | **NV_MRI_** | **WTV** |
| --- | --- | --- | --- | --- | --- |
| **Shapiro-Wilks test (p value)** | 0.006 | 0.012 | <0.001 | <0.001 | <0.001 |
| **Normality** | No | No | No | No | No |
| **Log transformation** | Yes | Yes | Yes | Yes | Yes |
| **wCV (%)** | 31.5% | 20.4% | 24.6% | 16.3% | 10.6% |
| **wCV 95% CI (%)** | (23.5%, 48.0%) | (15.3%, 30.4%) | (18.5%, 37.0%) | (12.3%, 24.0%) | (8.0%, 15.4%) |
| **RCln (%)** | 76.0% | 51.4% | 61.0% | 41.8% | 27.8% |
| **RC LOA**  **(RC_L_, RC_U_) (%)** | (-53.2%, 113.8 %) | (-40.2%, 67.2%) | (-45.7%, 84.1%) | (-34.1%, 51.8%) | (-24.3%, 32.1%) |

**Supplementary Table S3**. Results from repeatability assessment including results from Shapiro-Wilks test for normality and whether data were subsequently log-transformed to obtain wCV and RC data. wCV = within-subject coefficient of variation, 95% CI = 95% confidence intervals on wCV, RC = repeatability coefficient, LOA = limits of agreement, RC_L_ = RC lower limit of agreement, RC_U_ = RC upper limit of agreement.
