# Supplementary material for: Oxygen-Enhanced MRI Detects Incidence, Onset, and Heterogeneity of Radiation-Induced Hypoxia Modification in HPV-Associated Oropharyngeal Cancer
Source: Clin Cancer Res. 2024 Aug 9;30(24):5620–9. doi: 10.1158/1078-0432.CCR-24-1170 (PMC11654720; doi:10.1158/1078-0432.CCR-24-1170)
Supplement: Supplementary Table S4 — Summary of cohort lesion parameter median values at baseline and W2 and W4. [file ccr-24-1170_supplementary_table_s4_suppst4.docx]

| **Parameter** | **Baseline (BL)** **(Median, [IQR])**  **N = 36 lesions** | **W2**  **(Median, [IQR])**  **N = 33 lesions** | **p value** | **W4**  **(Median, [IQR])**  **N = 20 lesions** | **p value** |
| --- | --- | --- | --- | --- | --- |
| **ΔR_1_ (s^-1^)** | 0.018 [0.011, 0.026] | 0.030 [0.021, 0.039] | p < 0.001 | 0.031 [0.021, 0.043] | p < 0.001 |
| **HF_MRI_** | 0.42 [0.31, 0.50] | 0.28 [0.18, 0.42] | p < 0.001 | 0.33 [0.17, 0.39] | p = 0.001 |
| **HV_MRI_ (cm^3^)** | 11.3 [6.8, 28.3] | 6.9 [3.5, 13.0] | p < 0.001 | 5.9 [2.4, 8.6] | p < 0.001 |
| **NV_MRI_ (cm^3^)** | 19.8 [9.4, 37.8] | 18.0 [7.8, 46.5] | p = 0.126 | 12.8 [6.7, 20.6] | p < 0.001 |
| **WTV (cm^3^)** | 30.3 [19.3, 70.3] | 27.5 [11.8, 60.2] | p < 0.001 | 18.2 [10.0, 27.3] | p < 0.001 |

**Supplementary Table S4**. Summary of cohort lesion parameter median values at baseline and W2 and W4. The p-values (from mixed effects model) are for change from baseline to W2 or W4. The baseline (BL) value is the average if two baseline measurements (BL0 and BL1) were obtained.
